# Interfragmentary compression in a feline sacroiliac luxation model: biomechanical comparison of cannulated compression headless screws and cortical screws applied in positional or lag fashion

**DOI:** 10.1186/s12917-026-05388-w

**Published:** 2026-03-10

**Authors:** Laura Wachsmuth, Josef Wieser, Christoph Thorwächter, Yury Zablotski, Nina Dorothee Lorenz, Susanne Lauer, Matthias Kornmayer

**Affiliations:** 1 LMU Small Animal Clinic, Centre for Clinical Veterinary Medicine, Ludwig-Maximilians-Universität München, Munich, Germany; 2 Department of Orthopaedics and Trauma Surgery, Musculoskeletal University Center Munich (MUM), University Hospital, LMU Munich, Munich, Germany; 3 Northern Rivers Veterinary Specialists, Bangalow, Australia

**Keywords:** Cannulated compression headless screw, Position screw, Lag screw, Interfragmentary compression, Sacroiliac luxation fracture luxation, Cat, Feline

## Abstract

**Background:**

The purpose of this study was to compare the efficacy of cannulated compression headless screws (CCHS) with cortical positional and lag screws in generating interfragmentary compression in a simulated feline sacroiliac luxation fracture (SILF) model. In this ex vivo biomechanical study thirty-six cadavers of adult domestic short-haired cats with simulated unilateral SILF were allocated to three groups. Pressure mapping sensors were inserted into the joint space prior to fracture reduction. The SILF models were stabilized with 2.5 mm CCHS (*n* = 12), 2.4 mm cortical position (*n* = 12) or lag screws (*n* = 12), each spanning 60% of the sacral width. Total force (N), area of compression (mm^2^) and total pressure (N/mm^2^) were recorded and compared between the three groups. Pairwise comparison (Dunn for Kruskal-Wallis, Student’s t-tests for Fisher’s ANOVA) determined post-hoc differences between groups.

**Results:**

Total force (*p* = 0.23), area of compression (*p* = 0.62) and total pressure (*p* = 0.22) did not differ significantly between the three groups.

**Conclusion:**

No statistically significant differences in interfragmentary compression were observed among the three screw types under the specific ex vivo static testing conditions used in the feline SILF model. These findings challenge the current recommendation for using compression screws in feline SILF from a biomechanical perspective.

## Introduction

Pelvic fractures are common in cats and represent approximately one third (32%) of all fractures [1]. Among these injuries, sacroiliac luxation fractures (SILFs) occur most frequently, with unilateral lesions occurring more commonly than bilateral ones [1, 2]. Conservative management may be appropriate in selected cases [3]; however, surgical stabilization is indicated in non-ambulatory cats or those with intractable pain, neurological deficits, marked displacement, pelvic canal narrowing, or concurrent fractures of the weight-bearing axis [3]. Surgical intervention aims to alleviate pain, enable earlier return to ambulation, and preserve pelvic canal width [3–7].

Traditionally, unilateral SILFs have been stabilized using cortical positional or lag screws, with the current recommendation being the placement of one or two cortical screws in lag fashion [8]. Screws spanning approximately 60% of the sacral width are recommended to reduce the risk of implant failure [9]. These screws may be placed through an open approach or minimal invasively by fluoroscopic guidance, with the latter increasingly adopted in recent years [2, 8–14]. Fluoroscopic-assisted closed reduction and percutaneous fixation have improved screw placement accuracy, reduced screw loosening, and resulted in excellent functional outcomes [12–14]. Cannulated screws are typically used for these procedures because they can be advanced precisely over a guide wire [13, 14]. Cannulated cortical and headless compression screws (HCS) are available and their use has been reported in cats [10, 12–14]. Headless compression screws were introduced to achieve controlled interfragmentary compression by using threads of different pitch or diameter at the head and tip [15]. Since their development, a second generation of HCS has been designed to increase compressive strength [15–18]. In an experimental ovine humeral condylar fracture model, cortical lag screws demonstrated greater interfragmentary compression compared to cortical positional screws [19].

Interfragmentary compression is believed to enhance construct stability and promote fracture healing and is recommended for SILF stabilization [20, 21]. However, at the time of submission of this study, evidence regarding the compressive performance of currently available screw types in feline sacroiliac fixation is lacking.

The objectives of this study were to evaluate the efficacy of cannulated compression headless screws (CCHSs) to generate interfragmentary compression in a simulated feline sacroiliac luxation fracture model and to compare their performance with that of traditionally used cortical positional screws and recommended cortical lag screws.

Based on the improved design of second-generation HCSs, we hypothesized that a CCHS spanning 60% of the sacral width would achieve greater interfragmentary compression than either of the two cortical screw types. In addition, we hypothesized that the lag technique would produce superior compression compared with positional fixation using cortical screws.

## Materials and methods

### Specimens

Thirty-six cadavers of cats were used for this study. The use of the cadavers was approved by the Ethics Committee of the Faculty of Veterinary Medicine of the LMU Munich (AZ 413-22-08-24). The cats had been privately owned and were either deceased or euthanized for reasons unrelated to the study. Inclusion criteria were breed (DSH), skeletal maturity and normal pelvic structure. Orthogonal radiographs of each pelvis were obtained (Luminos dRF Max, Siemens Healthcare GmbH, Germany) and assessed to confirm skeletal maturity and the absence of orthopedic disease involving the hip or sacroiliac joint. Pelves without pathological or congenital abnormalities were dissected free of all soft tissues preserving the sacroiliac joint capsule within 24 h after decease or euthanasia. Following preparation, all pelves were wrapped in saline-soaked gauze and stored at -20° Celsius until testing.

### Radiographic determination of the screw length

The length of the screws was calculated by one board-certified surgeon (MK) on calibrated radiographs to span approximately 60% of the sacral width, as previously recommended [9]. For this purpose, the width of the sacrum, left ilium, and the left sacroiliac joint space were measured at the cranio-caudal midpoint of the sacral body as previously recommended [22].

### Groups

The pelves were randomly assigned using a software-based method (Excel, Microsoft Co.) to the following experimental treatment groups: cannulated compression headless screw (CCHS, *n* = 12), position screw (PS, *n* = 12) and lag screw (LS, *n* = 12). All surgeries were performed by one board-certified surgeon (MK).

### Implants

For the CCHS group, 2.5 mm short threaded, self-drilling, self-tapping, titanium alloy cannulated compression headless screws (DePuy Synthes, Johnson&Johnson, West Chester, Pennsylvania, USA) were used. For the position and lag screw groups, 2.4 mm fully threaded, self-tapping stainless steel cortical screws were used (DePuy Synthes).

### Drill-hole preparation

The specimens were thawed to room temperature for 24 h. The sacroiliac joint was left intact during drilling to ensure correct anatomical reduction. Therefore, pilot holes for both screw types were initiated using Kirschner wires (K-wire, Mede Technik GmbH, Emmingen-Liptingen, Germany). A 1.0 mm K-wire was selected because it can be placed more precisely than a larger-diameter K-wire. The entry points followed published recommendations [22] but were visually adjusted to account for individual anatomical variation. The K-wire was inserted from left to right and directed perpendicular to the long axis of the dorsal spinous process and parallel to the cranial endplate of the S1 vertebra, passing through the centre of the sacral body [22]. The K-wire was inserted to a depth shortly stopping after the spinal process. K-wire placement was assessed in orthogonal views via fluoroscopy (SIREMOBIL Compact L, Siemens AG, Germany). Once correct placement was achieved, the depth for insertion of the 1.0 mm K-wires was calculated with a direct measuring device for CCHS 2.5/3.0 mm (DePuy Synthes). The depth of the K-wire was confirmed or corrected corresponding to the radiographic calculations. For the CCHS, the K-wire was overdrilled using a 2.0 mm cannulated drill bit (DePuy Synthes). For the cortical screws, a cannulated 1.8 mm drill bit was not available. Therefore, the hole of the 1.0 mm K-wire was progressively enlarged using further K-wires of increasing sizes (1.2 mm, 1.4 mm, 1.5 mm) in order to preserve the correct position and thereby reduce the risk of drill bit deviation. Subsequently, the final hole was drilled using a 1.8 mm drill bit (DePuy Synthes). The correct localization of the drill bits were verified using fluoroscopy in orthogonal views. The depth for insertion of the enlarging K-wires and drilling was performed using marked reference K-wires and reference drill bits according to the initial measurement. Finally, for lag screws a gliding hole was drilled through the ilium with a 2.4 mm drill bit (DePuy Synthes).

### Specimen Preparation

Subsequently, the capsules of the left sacroiliac joint were disarticulated using a scalpel blade. The left pubis was transsected halfway between the pubic symphysis and the left ilio-pubic eminence using an oscillating saw (Colibri II, DePuy Synthes). The left ischium was transsected with the same saw, extending cranially from the left obturator foramen to the ischiatic arch caudally. The capsules of the right sacroiliac joint were disarticulated using a scalpel blade, and the right ilium was discarded. Finally, all pelves were wrapped in saline-soaked gauze and stored frozen (-20°) until testing.

### Insertion torque determination

For standardization, preliminary testing was performed in order to determine the average screw stripping torque for the cortical screws. The pelves of additional 10 cadaveric DSH cats, not assessed for interfragmentary compression, were used (three females, seven males, median body weight of 4.2 kg (range 2.3–6.2 kg). The animals met the same inclusion criteria as used for the specimens of the study and radiographic evaluation of the sacral width was performed in the same manner as described above. The pelves of five cats each were randomly allocated to cortical screws in a positional or lag fashion. The screw lengths were 22–24 mm, irrespective of technique used. The procedures of pre-insertion set up and final screw insertion was the same as outlined previously. The screws were inserted using a calibrated electronic torque screwdriver (ANPUDS^®^, *Hefei Zhidi Network Technology Co.*,* LTD*, China). The torque applied at the time of screw stripping was recorded. A mean stripping torque of 0.25 Nm for the position screws and 0.32 Nm for the lag screws was determined. According to the recommendations a torque value of 80% of the mean screw stripping torque was calculated and used as the target insertional torque for the two insertion techniques (0,20 Nm and 0,26 Nm) [23]. In accordance with the manufacturer’s recommendations, the CCHSs were inserted with all threads of the screw head fully engaged; therefore, insertion torque was not determined for this screw type. Applying a pre-determined insertion torque for CCHSs would have resulted in suboptimal screw positioning, as individual anatomical variations could have caused the screw to pass through the thin ilium.

### Pressure map testing

Pressure mapping sensors with a central hole for screw placement are available in limited sizes. The hole diameters of these sensors were too large for the implants assessed in this study and would have resulted in reduced amount of data acquisition. Consequently, these sensors were not used. Instead, the pressure Mapping Sensor 4000 (Tekscan, Inc., 333 Providence Highway, Norwood, MA 02062, USA) was used and holes matching the diameter of the screws (2.4 and 2.5 mm) tested were created in order to maximize data acquisition. In the preliminary tests the pressure mapping sensor was evaluated for optimal position to cover the greatest amount of joint surface, while allowing equal amounts of data acquisition surrounding the screw. Further, possible kinking of the sensor due to the irregular shape of the joint was assessed. Subsequently, several methods for screw hole creation were evaluated (e.g. drilling, use of a pointed chisel, or a hole chisel), but these approaches resulted in long cracks or oversized holes relative to screws diameter. Laser cutting may represent an alternative; however, it was neither available nor affordable for this study. Therefore, a machinery was developed to allow for standardization of localization and size of the holes in the sensor maps. The holes were induced thermally (using an iron cone at 300 °C for 3 s), which was found to have the least effect on structure, morphology and accuracy on mapped data outcome. Specifically, melting the hole margins using heat prevented cracking and preserved the structural integrity of the sensors. The disruption of the sensory matrix resulted in loss of a line and column of sensors. Among the available options, the introduction of the described perforation method resulted in the least alteration of the sensors and loss of data. The data loss is further partially compensated for by inquiring a sensor relative average algorithm of the deployed measurement software platform Tekscan I-Scan^®^ (Tekscan, Inc., 333 Providence Highway, Norwood, MA 02062, USA). Subsequently, the sensors were equilibrated and calibrated following the manufacturers pretesting regime for final suitability testing. The assessment revealed that this adjustment resembled the probed physical conditions.

### Screw placement

Before testing, the pelves were thawed to room temperature over 24 h. The sacral specimens were potted by incorporating approximately one third of the right sacrum into a metal device by using fast curing synthetic resin (SikaBiresin^®^ F180). After setting, the left ilium was reduced. The screws were inserted through the ilium as well as the hole of the pressure mapping sensor into the sacral body and hand-tightened using the corresponding screwdrivers for cortical screws combined with the calibrated electronic torque screwdriver regarding the prelimarily evaluated insertion torques for the cortical screws (0,20 Nm, 0,26 Nm). The CCHS (DePuy Synthes) were inserted the same manner using the corresponding screw driver and hand-tightened, such that all threads of the screw head were engaged.

### Biomechanical testing

**Fig. 1:**
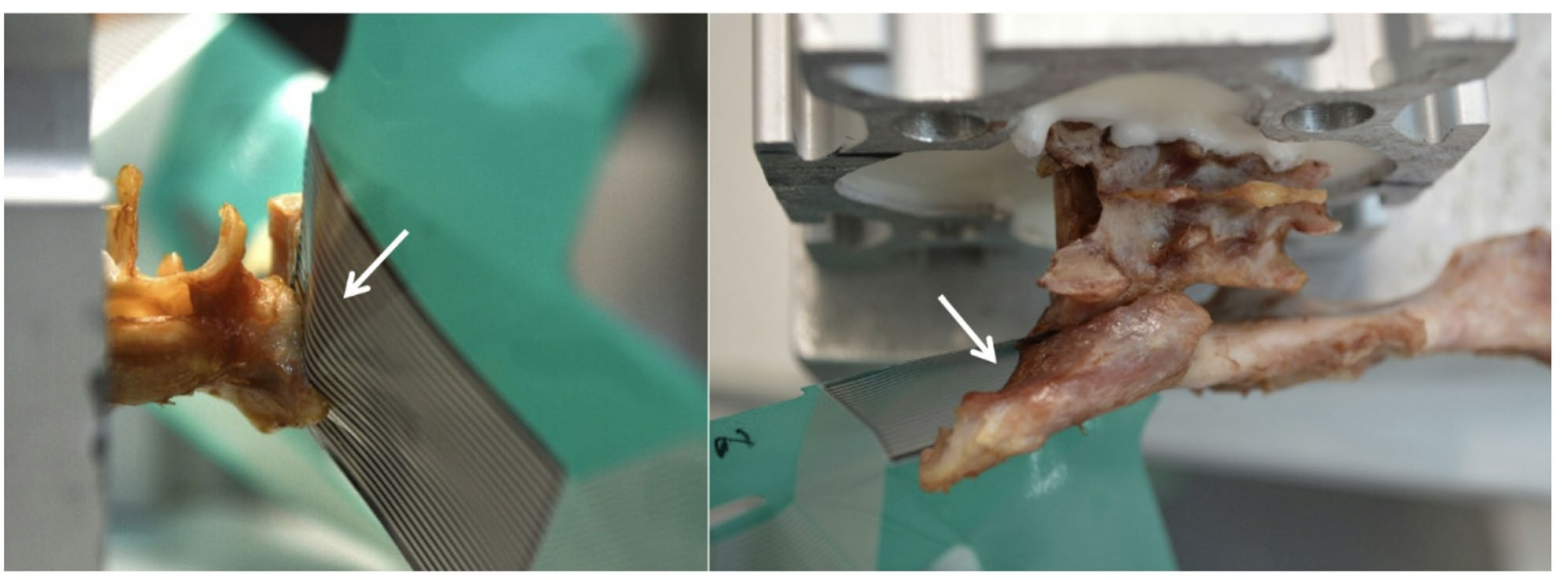
Photograph exemplarily representing a simulated sacroiliac luxation fracture specimen in a cat stabilized with a 2.4 mm cortical lag screw. Note the pressure mapping sensor (arrows) in the sacroiliac joint surrounding the screw

Data was recorded by the Tekscan I-Scan^®^ program (Tekscan, Inc., 333 Providence Highway, Norwood, MA 02062, USA) (Fig. 1). For comparison of the groups, the area of compression and interfragmentary compression was determined. Interfragmentary compression of the sacroiliac joint was calculated by total force in Newton (N) in relation to the total area of compression (mm^2^) and defined as total pressure (N/mm^2^). Operators were not be blinded to group allocation during data acquisition.

### Statistical analysis

The sample size of 12 specimens was determined in accordance with recommendations for pilot studies, which generally consider approximately 10–15 specimens per group to be adequate [24, 25]. Therefore, a power analysis was not performed.

Data were analyzed using commercial statistical software (R version 4.5.0 (2025-04-11)). Comparison of the three groups was performed for body weight, area of compression (mm²), total force (N) and interfragmentary compression (N/mm²). The normality of the data was assessed using the Shapiro-Wilk test. A normal distribution was present for body weight and area of compression. Consecutively, the Levene-test was used to test for homogeneity of variances. The variances tested homogeneous, and a Fischer-ANOVA was subsequently performed. Data for total force and total pressure were non-normally distributed. Therefore, the non-parametric Krustal-Wallis test was used for these data. Pairwise Dunn for Kruskal-Wallis and pairwise Student’s t-tests for Fisher’s ANOVA were performed to determine post-hoc differences between groups. A p-value < 0.05 was considered statistically significant, and values of 0.05 < *p* ≤ 0.10 were interpreted as indicating a statistical trend. Due to low number of observations no p-value correction for multiple comparisons was applied in order to reduce the type 2 error.

## Results

A total of 36 cadavers of domestic shorthair cats were analysed in this study. 19 cats were males and 17 were females. The mean age was 13.3 years (range 3–20 years). As in 12 cats the age was unknown, age was not analyzed statistically for differences between the groups. The mean body weight of 3.7 kg (range 2.3–7.2 kg). No significant differences were detected for the body weight (*p* = 0.73) between the groups, supported by a very small unstandardized effect of size (0.00). The lengths of the screws investigated ranged from 20 to 26 mm in all three groups (CCHS, PS, LS).

**Fig. 2:**
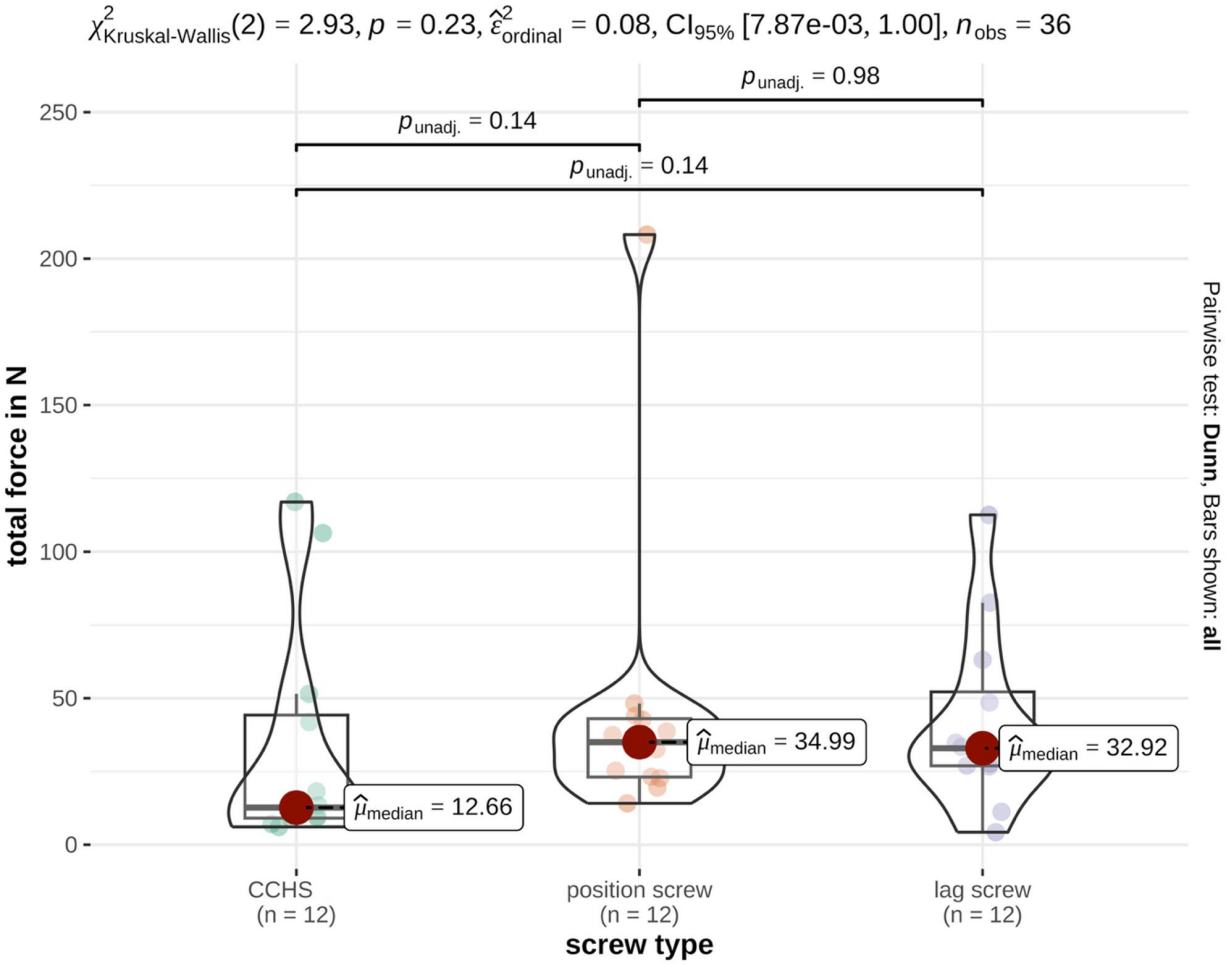
Illustration of the median values of total force (N) generated by the three groups tested in a feline cadaveric model of SILF. No significant differences were found between the groups

For total force, the following median values were obtained: CCHS, 12.66 N (IQR 35.20); PS, 34.99 N (IQR 19.93); LS, 32.93 N (IQR 25.24) (Table 1). No significant difference was observed among the three screw types (*p* = 0.23), with a medium unstandardized effect size (0.08) (Fig. 2). Detailed statistical information is provided in Table 2.

**Table 1:** Demonstration of the compressive forces (median values, interquartile range) and the areas of compression (mean values, standard deviation) of the three groups tested in a feline cadaveric model of SILF

	Total force (*N*)	Area of compression (mm^2^)	Total pressure (*N*/mm^2^)
CCHS	12.66 (35.20)	69.00 ± 23.07	0.24 (0.37)
position screw	34.99 (19.93)	78.75 ± 23.29	0.48 (0.27)
lag screw	32.92 (25.24)	76.00 ± 28.09	0.40 (0.37)
*p*-value	0.23	0.62	0.22

No significant differences were present between screws

**Table 2:** Range, mean, confidence intervals, unstandardized effect size, median, and standardized effect size for the areas of compression and compressive forces in the three groups tested in a feline cadaveric model of SILF. PS = positional screw; LS = lag screw

Screw type	Variable	Range	Mean [95% CI]	Cohen’s d Interp (d)	Median [Q1-Q3]	Rank Biserial Interp (RB)
CCHS 60%	area (mm^2^)	24–108	69 [54.3, 83.7]	-0.23 small	65 [54.2–84.5]	-0.17 small
CCHS 60%	total force (N)	6-117	33.4 [8.4, 58.4]	-0.18 very small	12.7 [9.1–44.3]	-0.35 large
CCHS 60%	total pressure (N/mm^2^)	0.1–1.1	0.4 [0.2, 0.6]	-0.24 small	0.2 [0.2–0.5]	-0.36 large
PS	area (mm^2^)	52–135	78.8 [64, 93.5]	0.17 very small	76.5 [64-84.5]	0.14 small
PS	total force (N)	14.1-208.2	46.4 [13.3, 79.5]	0.14 very small	35 [23.1–43]	0.18 small
PS	total pressure (N/mm^2^)	0.3–1.5	0.5 [0.3, 0.7]	0.12 very small	0.5 [0.3–0.6]	0.19 small
LS	area (mm^2^)	32–145	76 [58.2, 93.8]	0.06 very small	73 [64.5–83.2]	0.03 tiny
LS	total force (N)	4.3–112.6	42 [22.5, 61.5]	0.03 very small	32.9 [26.9–52.2]	0.17 small
LS	total pressure (N/mm^2^)	0.1–1.1	0.5 [0.3, 0.7]	0.11 very small	0.4 [0.3–0.7]	0.17 small

For area of compression, the following mean values were obtained: CCHS, 69.00 mm^2^ (SD ± 23.07); PS, 78.75 mm^2^ (SD ± 23.29); LS, 76.00 mm^2^ (SD ± 28.09). No significant difference was observed across the three screw types (*p* = 0.62), with a very small unstandardized effect size (0.00) (Table 1). Detailed statistical information is provided in Table 2.

**Fig. 3:**
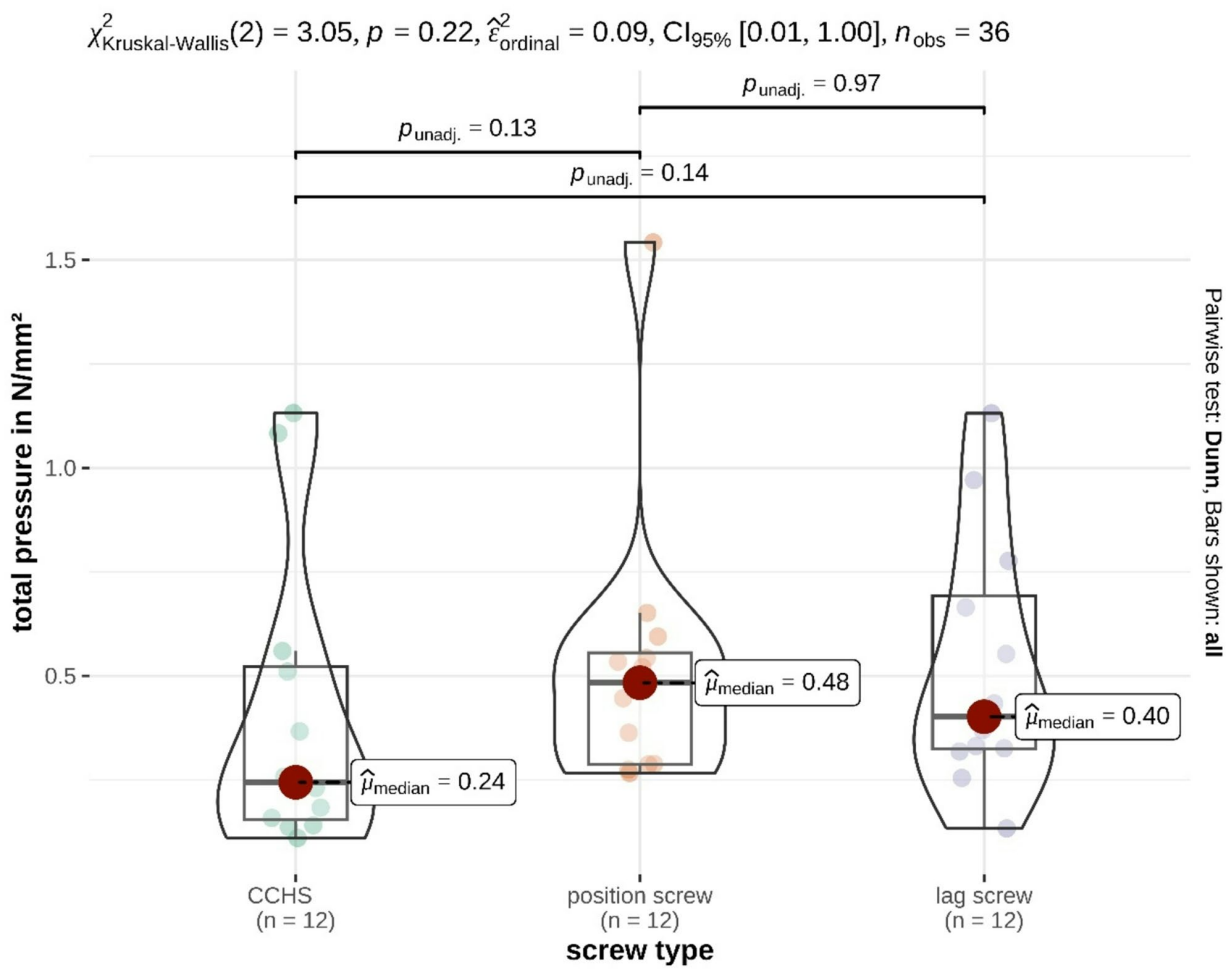
Illustration of the median values of total pressure (N/mm^2^) generated by the three groups tested in a feline cadaveric model of SILF. No significant differences were found between the groups

For total pressure, the following median values were obtained: CCHS, 0.24 N/mm² (IQR 0.37); PS, 0.48 N/mm² (IQR 0.27); LS, 0.40 N/mm² (IQR 0.37) (Table 1). No significant difference was observed among the three screw types (*p* = 0.22), with a medium unstandardized effect size (0.09) (Fig. 3). Detailed statistical information is provided in Table 2.

Although, no statistical trend (0.05 < *p* ≤ 0.10) was observed, p-values between the CCHS and cortical screw groups (*p* = 0.13–0.14) may indicate small to moderate differences that were not detected because of the limited sample size (*n* = 12). A post hoc power analysis yielded a power of 23% for total pressure (N/mm^2^), indicating that approximately 42 specimens per group would be required to achieve 80% power and reach statistical significance.

## Discussion

It was hypothesized that CCHSs would generate greater interfragmentary compression than cortical screws, and that screws inserted in a lag fashion would outperform positional screws. However, in this cadaveric feline SILF model, all three screw types produced comparable interfragmentary compression, and no statistically significant differences were identified. Consequently, both hypotheses were rejected.

This study demonstrated that CCHSs achieved compressive forces comparable to those generated by cortical screws. Headless compression screws are designed to enhance interfragmentary compression through differently inclined threads at the head and the screw tip [15–18], which is thought to improve fixation rigidity [20]. Interestingly, this effect was not observed in the present cadaveric feline SILF model. Several factors related to screw design and insertion technique, including outer diameter and drill bit size, may have contributed to these findings. However, the threaded configuration of the screws – partially threaded CCHSs versus fully threaded cortical screws – may represent the most influential factor. In general, lag screws generate compression across the fracture gap when the threads engage the far fragment and the second cortex [26–28]. In SILFs, screws are ideally inserted to engage approximately 60% of the sacral width, resulting in a screw tip that is centrally positioned within the sacrum. This region consists predominantly of cancellous bone, and the screw does not engage the far cortex. As a result, partially threaded screws engage only cancellous bone, depending on the length of the threaded part. In contrast, fully threaded screws engage bone along their entire length, including the abaxial cortex of the sacrum. The authors hypothesize that the reduced cumulative bone-implant interface of CCHSs may explain their performance in the present study.

This study showed that cortical positional screws generated compressive forces comparable to those inserted in a lag fashion. This finding was unexpected and may also be explained by differences in the amount of bone engaged. Theoretically, lag screws generate compression between two fragments, while positional screws primarily maintain fixation [26–28]. However, a previous study evaluating cortical positional and lag screws in an ovine humeral condylar fracture model demonstrated that both techniques initially generated compressive forces during insertion [19]. During screw insertion, tightening torque is converted into axial compression along the screw shaft via threads [28], meaning that compressive load is generated primarily by the threads rather than the screw head [19]. In the aforementioned ovine study, cortical lag screws ultimately generated significantly higher compressive forces than positional screws, which was attributed to a more homogenous distribution of compressive load via the screw head [19]. This contrasts with the findings of the present study and may be explained by anatomical differences between the cadaveric models. In the current investigation, the only difference between the insertion techniques of the two cortical screws was the presence of a glide hole. Consequently, positional screws engaged additional cortices of the ilium. Therefore, the comparable performance of positional screws observed in the present study may be attributed to a larger cumulative bone-implant interface, which results in increased axial load capacity generated by the screw threads during insertion. Another possible explanation for the similar performance of the two cortical screw types observed in the present study is that washers were not used with the cortical lag screws. In fracture fixation, washers may be applied in regions where cancellous bone is covered by a thin cortical layer [28]. Washers help distribute the load beneath the screw head and can thereby increase compression in fracture models [29]. Consequently, the use of a washer with cortical lag screws might have resulted in different compressive forces. In summary, the exact mechanical mechanism underlying these effects cannot be definitively established, and the role of washers in feline SILF has not yet been determined. Overall, further biomechanical studies are required to clarify the findings of the present study.

The findings of this study challenge the current recommendation that advocate the use compression screws and cortical lag screws without washers for the management of feline SILF from a biomechanical perspective. Under the specific ex vivo static testing conditions used in this feline SILF model, no biomechanical advantage of this implants was demonstrated. Considering the anatomical and mechanical factors discussed, sacroiliac joint stabilization may not be directly extrapolated from general fracture fixation principles. As no statistically difference was identified between fully threaded cortical positional screws and compression screws in this cadaveric feline SILF model, interfragmentary compression may not be biomechanically necessary in feline SILF. Nevertheless, this interpretation requires further biomechanical validation, as previously discussed.

This study has several limitations. Static, single-point compression testing does not replicate in vivo conditions, and dynamic contact loading may better reflect clinically relevant screw performance [30].

Considerable data variability was observed. As the methodology was consistent, biological heterogeneity, such as differences in bone quality among specimens, is a likely explanation. Because medical histories were incomplete, potential factors affecting bone quality, such as endocrinological diseases, could not be evaluated. In addition, the mean age of the cats was higher than that reported in clinical studies of HCSs [13, 14], supporting this interpretation. Insertion torque differed between cortical lag and positional screws, consistent with findings from a canine biomechanical study [31] and possibly attributable to differences in bone density. All specimens underwent two freeze-thaw cycles for organizational reasons, which might have influenced compressive force measurements; however, a previous work suggests that multiple freeze-thaw cycles do not alter biomechanical properties [32]. Pre-testing assessment of bone density and specimen stratification may therefore have reduced variability [33].

Interfragmentary compression was assessed using a pressure-mapping sensor within the sacroiliac joint, which may not adequately represent the joint surface. The sacroiliac joint is composed of fibrocartilage and fibrous tissue and is non-planar [22], with substantial interindividual variation in size, shape, and articular morphology [34]. Although, no significant differences in contact area were detected between groups and effect sizes were minimal, the inherent stiffness of the sensor introduces additional resistance [19] and limits its ability to conform to joint anatomy. Consequently, the measurements provide only an indirect estimate of force distribution and do not fully reproduce in vivo conditions.

Because no prior data was available for power calculation, this investigation was designed as a pilot study and sample size was based on published recommendations [24, 25]. No statistical trends were observed. However, p-values between the CCHS and cortical screw groups suggest small to moderate differences that may not have been detected due to the limited sample size. Post hoc power analysis indicated low statistical power, with substantially larger group sizes required to achieve adequate power. Increasing specimen numbers would likely clarify differences between CCHSs and cortical screws and yield more clinically relevant results.

Overall, these limitations restrict direct extrapolation of the findings to clinical implant performance.

## Conclusion

No statistically significant differences in interfragmentary compression were observed among the three screw types under the specific ex vivo static testing conditions used in the feline SILF model. These findings challenge the current recommendation for using compression screws in feline SILF from a biomechanical perspective. However, dynamic loading studies are needed for a more comprehensive evaluation of compressive screw performance.

## Data Availability

The datasets analyzed during the current study are available from the corresponding author on reasonable request.
